# Antioxidant effects and protective potential of fruit extracts of *Detarium microcarpum *against arsenic trioxide-induced human lymphocytes DNA oxidative damages

**DOI:** 10.22038/AJP.2024.24842

**Published:** 2025

**Authors:** Ablassé Rouamba, Vincent Ouedraogo, Maurice Ouédraogo, Martin Kiendrebeogo

**Affiliations:** 1 *Laboratoire de Biochimie et Chimie Appliquées (LABIOCA), UFR-SVT, Université Joseph KI-ZERBO, 03 BP 7021, Ouagadougou 03, Burkina Faso*; 2 *Laboratoire de Physiologie Animale (LAPA), UFR-SVT, Université Joseph KI-ZERBO, 09 BP 848, Ouagadougou 09, Burkina Faso*

**Keywords:** Arsenic trioxide, Detarium microcarpum, DNA repair, Phytomolecules

## Abstract

**Objective:**

The integrity of the DNA is continously menaced by the harmful genotoxic compounds. The endogenous system responsible for preserving the DNA integrity, often fails following a massive influx of these

genotoxic compounds. Reseaches on exogenous bioactive compounds from fruits and vegetables are necessary. This study was designed to evaluate the free radical scavenging activity and the DNA protection/repair potentiality of the extracts of *Detarium microcarpum *fruit pulp to protect against the arsenic trioxide-induced DNA oxidative degradation.

**Materials and Methods:**

The ability of extracts to trap free radicals was assessed by using the 2,2-diphenyl-1-picrylhydrazyl (DPPH), nitric oxide and hydroxyle radicals quenching assay. The comet assay was performed for evaluating the DNA protection/repair property of extracts to inhibit the DNA oxidative damage induced by arsenic trioxide.

**Results:**

All extracts at a final concentration of 50 µg/mL have quenched more than 50% of DPPH, nitric oxide and hydroxyle radicals. Moreover, all extracts have showed good DNA protection/repair activity against the arsenic trioxide-induced DNA oxidative damage compared to arsenic treatment alone (p<0.001). However, methanol fractions have exhibited the best DNA protection/repair activities by reducing considerably DNA fragmentations compared to arsenic treatment (p<0.001). The genoprotective activity of the extracts was positively correlated with their free radical scavenging abilities.

**Conclusion:**

The methanol fraction of *D. microcarpum* fruits have exhibited interesting DNA protection /repair properties probably due to its free radicals quenching ability. Further investigations are necessary to identify the phytomolecules responsible for these biological activities.

## Introduction

Reactive oxygen species (ROS) and reactive nitrogen species (RNS) are generated by the normal metabolism of aerobic organisms (Arfin et al., 2021). These reactive species can also be generated following exposure to exogenous genotoxins such as environmental pollutants (ionizing radiation, heavy metals, radioactive compounds, hydrocarbons). During chronic oxidative stress, ROS can cause severe damage to biological macromolecules including nucleic acids, membrane lipids and proteins (Sanna and Fadda, 2022). The oxidative degradations of biological macromolecules are associated to the development or aggravation of many pathologies such as neurodegenerative diseases (Alzheimer's and Parkinson's diseases), diabetes, obesity, cardiovascular diseases and cancer (Liguori et al., 2018; Vona et al., 2021). 

Arsenic trioxide is a genotoxic agent resulting from environmental pollution. It is a major contaminant of several groundwater (Bellamri et al., 2018). The genotoxicity of arsenic trioxide involves two mechanisms: an increase in the intracellular level of ROS following its deleterious action on antioxidant enzymes and a suppression of the endogenous DNA damage repair system (Faita et al., 2013; Dashner-Titus et al., 2020). Previous studies demonstrated that arsenic trioxide causes normal peripheral blood lymphocytes death through mitochondrial pathway via enhancing oxidative stress (Zarei et al., 2019). Under oxidative stress conditions, when the endogenous systems for preserving DNA integrity prove insufficient to fight against the harmful effects of ROS and other genotoxic agents, it is then primordial to help the cells by supply of exogenous bioactive compounds from fruits and vegetables. 

Fruits and vegetables are a good source of health-promoting bioactive compounds (Li et al., 2016). Indeed, epidemiological studies have shown that there is a correlation between the consumption of foods rich in polyphenols (fruits and vegetables) and a lower risk of the onset of age-related diseases such as neurodegenerative diseases and cancers (Obeng et al., 2020). This relationship is often attributed to the potent antioxidant flavonoids and other polyphenols which have redox properties to directly remove reactive oxygen species. These compounds could also induce the expression of genes coding for antioxidant, DNA protection/repair enzymes (Cas and Ghidoni, 2018).

*Detarium microcarpum *Guill. et Perr. is a fruit plant of the caesalpiniaceae family. It is well known in West Africa for the highest nutritional value and the multiple medicinal properties of its fruit pulp (Adebayo et al., 2019). Previous studies have shown that the crude ethanolic extract of the fruit pulp of *D. microcarpum* protects the DNA of NMRI mice *in vivo* against the genotoxic effects of cyclophosphamide (Rouamba et al., 2017). The same extract showed a protective potential of human lymphocytes against the cytotoxicity of hydrogen peroxide and tert-butyl hydroperoxide (Rouamba et al., 2018). 

This study aims to evaluate *in vitro *the ability of the fractions of *D. microcarpum*’s fruits extracts to preserve the integrity of the human lymphocyte DNA against the arsenic trioxide known as a potent genotoxic agent. 

## Materials and Methods

### Chemicals

Ethylene diamine tetra acetic acid, 2,2-diphenyl-1-picrylhydrazyl (DPPH), 2-desoxy-D-ribose, hydrogene peroxide, nitroprusside sodium, ficoll-1077, Roswell Park Memorial Institute medium (RPMI-1640), Phosphate buffer saline, dimethylsulfoxide, triton-X, hank balanced Salt Solution, sodium hydroxide, sodium chloride, trizma base, propidium iodide, peniccilin-streptomycin, fetal bovin serum were purchased from Sigma (Steinheim, Germany). Thiobarbituric acid, arsenic trioxide were purchased from Fluka chemie (Buchs, Switzerland). Methanol, ethanol, ethyl acetate were purchased from Prolabo (France).

### Plant material and extraction

**Fruit collection**

 The fruits of *Detarium microcarpum* Guill and Perr were collected in Gampèla, a locality situated at 15 km from Ouagadougou East (12° 25′ 45″ N, 1° 23′ 20″W) in January 2013. A sample of the leafy stem was authenticated by Professor MILLOGO Jeanne, a botanist at the Université Joseph KI-ZERBO. A specimen of the leafy stem was deposited in the herbarium of the UFR/SVT (Université Joseph KI-ZERBO) under the identification code CI: 15928. The fruit pulp was first scraped manually and dried in the laboratory conditions (under ventilation at an ambient temperature of approximately 30° C, and protected from sunlight). The fruit pulp was then powdered and the powder obtained was finally stored in sachets of freezing for the extraction of the herbal drug.

### Extraction of the herbal drug

 The powder from the fruit pulp of *D. microcarpum* underwent a maceration in absolute ethanol at a ratio of 1:5 (Mass/Volume) for 24 hr under mechanical stirring. The macerated has been filtered. The filtrate was concentrated in an evaporator and evaporated to dryness. The crude ethanolic extract obtained was adsorbed in silica (Silicagel® 60, Merck, particle size 0.2-0.5 mm) and extracted successively with pure ethyl acetate, ethyl acetate/ methanol (1:1 v/v) and methanol as described previously (Carole et al., 2020). Three types of extract were obtained: ethyl acetate extract, ethyl acetate/methanol extract (1:1 v/v) and methanol extract.

### Free radical quenching assay

**DPPH radical scavenging assay**

 The anti-radical activities of the extracts were evaluated by using the DPPH (2,2-diphenyl-1-picrylhydrazyl) scavenging method as described previously (Baliyan et al., 2022). The extracts (50 μg/mL final concentration in the well) were mixed with a purple solution of DPPH (20mg/L in methanol) then incubated at room temperature for 15 min. The decolorization of the DPPH radical was monitored at 517 nm using a UV-Visible spectrophotometer (epoch 251465, Biotek Instruments, U.S.A.). The DPPH antiradical activity was expressed as a percentage relative to the control without extracts and containing DPPH and the solvent.

### Hydroxyle radical quenching assay

The ability of the fruit pulp extract to scavenge the hydroxyl radical was evaluated by using the deoxyribose degradation inhibition test as described previously (Tijani et al., 2018). The reaction mixture consisted of 100 μL of extract (final concentration of 50 μg/mL in 50 mM phosphate buffer, pH 7.4), 100 μL of EDTA aqueous solution (1.04 mM), 100 μL of iron Fe ^2+ ^(100 mM), 100 μL of deoxyribose (60 mM), and 100 μL of hydrogen peroxide (10 mM). The mixture was incubated (37°C for 1 hr) then 2 mL of trichloroacetic acid (15%) - thiobarbituric acid (0.675%) were added. The mixture was returned to incubation (100°C for 15 min). The scavenging of the hydroxyl radical was measured with a spectrophotometer at 532 nm against a blank (containing neither hydrogen peroxide nor iron sulphate). The activity of the extracts to scavenge the hydroxyl radical was expressed as percentage of inhibition of the deoxyribose degradation compared to the control without extract.

### Nitric oxide radical scavenging assay

The capacity of the extracts to trap the nitric oxide radical was evaluated according to the method described previously (Hellal et al., 2020). The extracts (final concentration of 50 μg/ml) were incubated (2 hr, room temperature) in the presence of sodium nitroprusside (10 mM). The reduction of the nitric oxide radical by the extracts was measured with a spectrophotometer at 550 nm after addition of Griess' reagent. The activity of the extracts to scavenge the nitric oxide radical was expressed as percentage of nitric oxide radical scavenging compared to the control without extract.

### DNA protection and DNA repair assay

**Lymphocyte isolation and culture**

A verbal agreement from the voluntary donors was obtained before the blood sample was taken. Authorization was obtained by the administration of “Centre Régional de Transfusion Sanguine (CNTS) de Ouagadougou (N° 2015-340/MS/SG/CNTS/CRTS-O). The blood was handled with respect for art and according to the codes of ethics of the declaration of Helsinki (version revised during the 64th General Assembly of the Wold Medical Association, Fortaleza, Brazil, October 2013). Lymphocytes were isolated from whole blood according to the procedure described previously with slight modifications (Puleo et al., 2017). Blood (5 ml) was mixed with an equal volume of phosphate buffer (pH 7.4) in a conical tube. The mixture was slowly poured over 3 ml of a ficoll solution into another conical tube and centrifuged (800 g at 4°C for 30 min). Lymphocytes were collected at the plasma-ficoll interface in a conical tube, washed three times with phosphate buffer by centrifugation (400 g at 4°C for 10 min. Cells were suspended in RPMI-1640 culture medium containing 20 mM glutamine, 10% fetal calf serum supplemented with 1% penicillin-streptomycin and incubated at 37°C in a 5% CO2 / 95% air atmosphere for 72 hr. The culture medium is renewed every 24 hours.

### Cell processing and comet assay

Cells were harvested by trypsinization for genoprotection and DNA repair assays. To assess the capacity of the extracts to protect DNA against arsenic trioxide, the cells (5000 cells/mL of culture medium) were previously brought into contact with the different extracts (50 μg/mL in culture medium) before being exposed to a solution of arsenic trioxide (500 µM) for one hour in an incubator (5% CO2; T°=37°C). A well containing the cell suspension and arsenic trioxide served as a positive control, and a well containing only the cell suspension and culture medium (RPMI) served as a vehicle. To assess the ability of the extracts to induce the DNA damaged repair, DNA degradation was firstly induced by exposing the cells to arsenic trioxide for 1 h, and the different extracts (50 μg/mL in culture medium) were then added to the cell suspension and incubated for 30 minutes. A well without extract but containing RPMI serves as a control. At the end of the different incubation’s times, the cells were harvested for the quantification of DNA damage by using the standard comet assay (Koppen et al., 2017). DNA damages were in first time evaluated by the DNA (comet) morphological analysis at 400x magnification using a fluorescence microscope (Carl Zeiss, Germany), and in second time the amount of DNA degradation was quantified by analyzing the comets with a CometScore software (version 1.5, TriTek corporation) and expressed as comet tail length, percentage of DNA in tail (%), tail moment and Olive moment (arbitrary unit).

### Statistical analysis

All results were expressed as mean value of several independent experiments (n=6) ± standard deviation (SD). One-way ANOVA followed by the Newman Keuls test were used to measure the degree of statistical significance of the data. A significant difference was considered for p<0.05.

## Results

**Free radicals scavenging activities**

**Figure 1:**
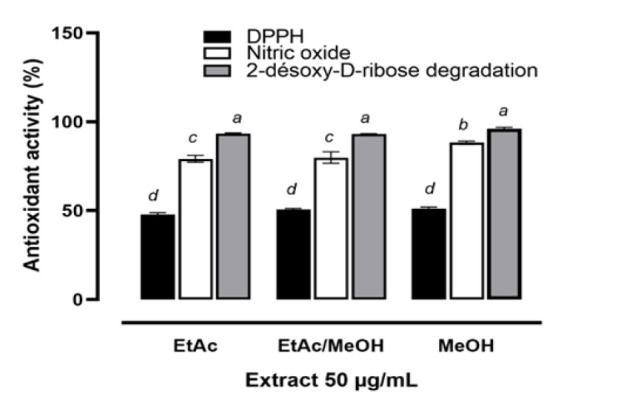
Free radical scavenging activity of *D. microcarpum *extract ; Values are expressed as Mean±SD (n=6). Different uperscript letters (^a,b,c,d^) on histogram indicate statistically different values (p<0.05). EtAc : Ethyl acetate ; MeOH : Methanol

The ability of the extracts to scavenge free radicals was evaluated using the DPPH radical, the nitric oxide radical and the hydroxyl radical (inhibition of 2-deoxy-D-ribose degradation) assays. Regarding to the Figure 1, all extracts at the final concentration of 50 µg/ml trapped more than 50% of DPPH, nitric oxide and hydroxyl radicals. Free radical scavenging activities are much better on nitric oxide and hydroxyl radicals. 

The extracts showed similar activities on DPPH radical scavenging and 2-deoxy-D-ribose degradation inhibition (p>0.05) on the other hand the methanolic extract showed the best nitric oxide radical scavenging activity (p<0.05).

**DNA protection and DNA repair activities**

**Figure 2:**
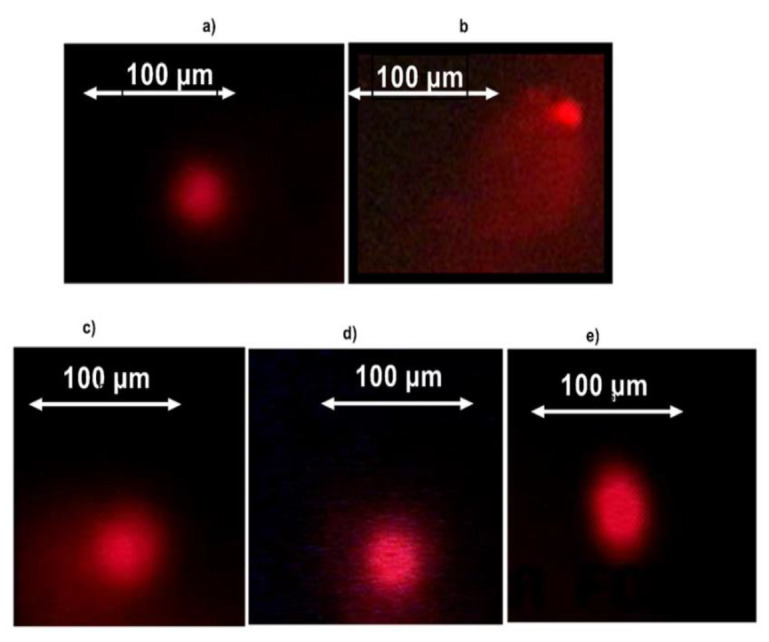
Image of comet observed at 400× magnification with a fluorescence inverted microscope (Carl Zeiss,Germany), ; a) vehicle (RPMI group) ; b) Arsenic trioxide alone ; c) Arsenic oxide + ethyl acetate extract ; d) Arsenic trioxide + ethyl acetate/Methanol (1 :1v/v) extract ; e) arsenic trioxide + methanol extract

The ability of the extracts to protect DNA against arsenic trioxide induced oxidative damage was firstly evaluated on human lymphocytes by comet morphological analysis. Considering the Figure 2, when the lymphocytes are treated with the Roswell Park Memorial Institute medium (RPMI-1640), the DNA after migration appeared in the form of a ball suggesting that the DNA is undamaged (Figure 2a). However, when the lymphocytes are treated with arsenic trioxide alone (genotoxic compound), the DNA after migration appeared in the form of a comet with a much more spread-out tail (Figure 2b) signifying severe degradations of the DNA caused by arsenic trioxide. The prior treatment of lymphocytes with different extracts before their exposure to arsenic oxide showed less DNA damages (Figure 2c, d,e) compared to DNA treated only with arsenic trioxide.

**Figure 3:**
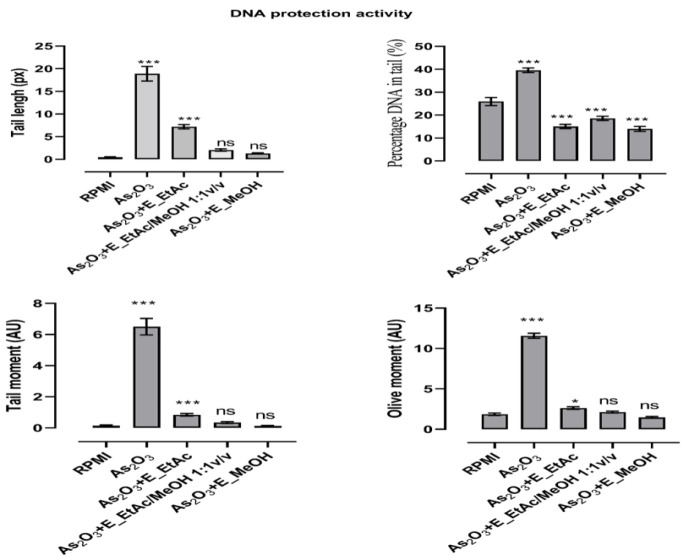
Protective activity of *D. microcarpum* extracts on arsenic trioxide induced human lymphocyte DNA damage ; Cells were exposed concomitantly to arsenic trioxide (500 µM) and extracts (50 µg/ml) the results are expressed as mean±SD (n=6), ^***^p<0.001; ^*^p<0.05 significant difference from the vehicle (RPMI group) ; ns : no-signifiacant différence from the vehicle)

**Figure 4:**
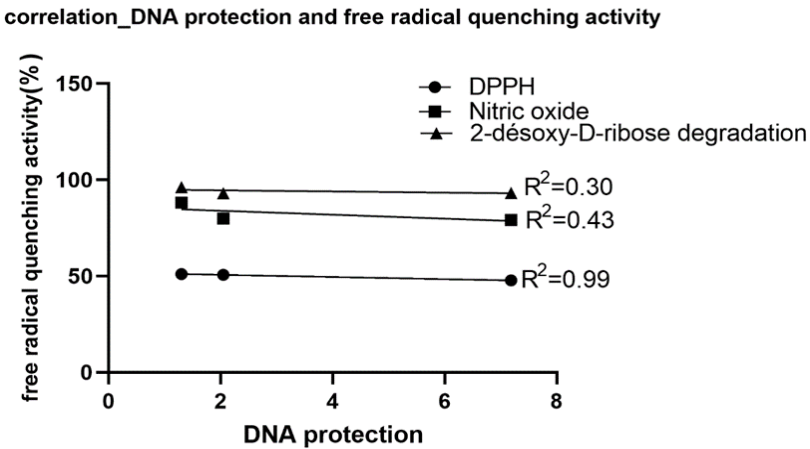
Contribution of the free radical scavenging ability of extracts to the DNA protection property

DNA damage was secondary quantified by considering comets parameters such as tail length (px), percentage DNA in tail (%), tail moment and Olive moment (arbitrary unit) by using the CometScore software (version 1.5, TriTek corporation) (Figure 3). Whatever the parameter used, arsenic trioxide induced very significant DNA degradation compared to RPMI culture medium (p<0.001). However, the extracts considerably reduced the genotoxic effects of arsenic trioxide when the cells are pretreated with the extracts before their exposure to the genotoxic compound. The best genoprotective activities were observed with ethyl acetate/methanol (1:1 v/v) and methanol extracts which totally inhibited the genotoxic effects of arsenic trioxide when tail length, tail moment and olive moment were considered. The ethyl acetate extract, although less active than the other extracts, significantly inhibited the genotoxic effects of arsenic trioxide (p<0.05). The free radical scavenging activities are positively correlated with the genoprotective activity of the extracts (Figure 4). The best correlation was obtained with the DPPH radical scavenging activity (R^2 ^= 0.99).

**Figure 5:**
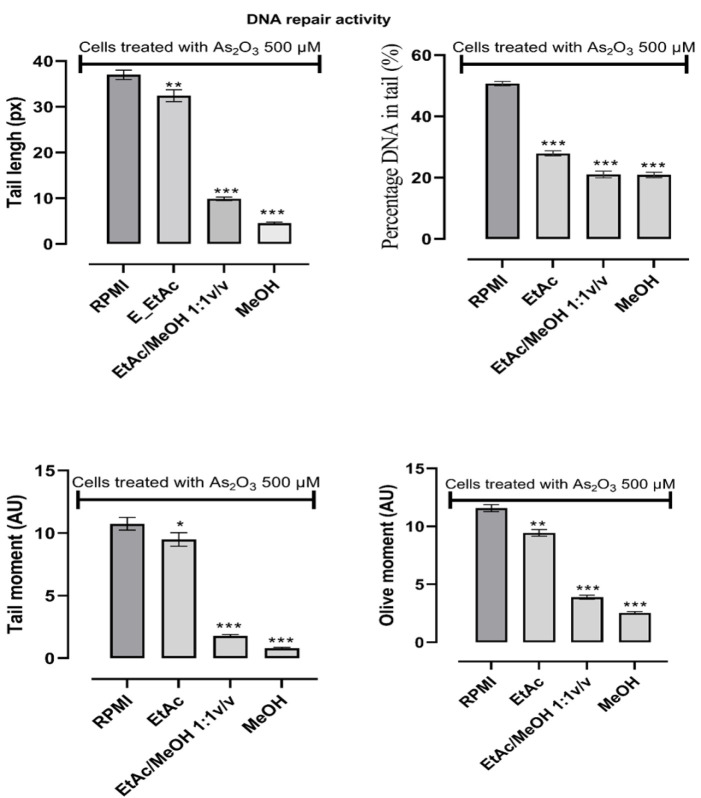
DNA repear activity of *D. microcarpum* extracts on arsenic trioxide induced human lymphocyte DNA damage ; DNA degradations were induced with As_2_O_3_ 500 µM for 1 hr prior to the extracts addition (50 µg/ml). The results were expressed as mean±SD (n=6), ^***^p<0.001; ^**^p<0.01 ; ^*^p<0.05 significant difference from the vehicle (RPMI group)

To measure the ability of the extracts to induce the endogenous DNA damage repair system, the cells were first exposed to arsenic trioxide before the addition of the extracts and the DNA degradations were recorded (Figure 5). Whatever the parameter considered to quantify the DNA damage, all the extracts showed good DNA repair activities when the DNA damage of the cells of the control group (cells exposed to arsenic trioxide and of the culture medium) are compared with the DNA damage of cells previously exposed to arsenic trioxide before being treated with extracts (p<0.05). The methanolic extract showed the best DNA repair activity.

## Discussion

In view of the demonstrated biological activities, the extracts of D. microcarpum would preserve the integrity of genomic DNA against genomic damages induced by arsenic trioxide. The extracts showed in this study good antioxidant activities through the inhibitions of 2-desoxy-D-ribose, DPPH and nitric oxide radicals that could justify their genoprotective property (Gafrikova et al., 2014). ROS/RNS (hydroxyl radical and nitric oxide) produced by cell metabolism under oxidative stress condition caused biomolecules damages such as lipids peroxidation, toxic aldehydes formation, DNA strand breaks and DNA-protein adducts. Lipid peroxidation compromises membrane integrity and triggers programmed cell death through the apoptosis pathways activation (Bhagyanathan and Thoppil, 2016). Moreover, the OH produced by the hydrogen has highly affinity to DNA causing strand breakage. This process can result in DNA instability, mutagenesis and ultimately carcinogenesis or cell death (Boligon et al., 2012). Indeed, the extracts, by trapping free radicals, would prevent the direct interaction of these radicals on the DNA molecule and therefore reduce DNA strand breaks. Furthermore, the extracts could induce the endogenous antioxidant gene (1st line of DNA protection) expression. Indeed, endogenous antioxidant enzymes such as catalase, glutathione peroxidase and oxide dismutase protect cellular biomolecules (DNA, proteins and lipids) against oxidative degradation (Muenyi et al., 2015; Stevens et al., 2018). Trapping directly the free radicals generated during the metabolism of arsenic trioxide, fruit extracts could reduce oxidative damage to DNA, in particular those caused by hydroxyl radicals and nitric oxide. In the second pathway of the arsenic trioxide genotoxicity, it interferes with the DNA repair system and leads to a mutation of tumor suppressor genes, in particular the protein P53, involved in the regulation of the cell cycle and in the repair genomic damage. The fruit extracts showed in this study good restorative activities of DNA damage induced by arsenic trioxide. Fruit extracts could induce the intrinsic cellular repair system activation by enhancing the protein P53 gene expression, thus restoring the integrity of DNA (Chatterjee and Walker, 2017; Shrinivas et al., 2017). Previous phytochemical investigations of the fruit pulp of D. microcarpum showed very high contents of total flavonoids and total phenolics which would be responsible for the DNA repair activities recorded in this study (Lamien-Meda et al., 2008). Indeed, antioxidant compounds like flavonoids due to their redox potential can neutralize free radicals (hydroxyl radical, oxide nitric radical) by a mono-electronic transfer, impeaching their interaction with DNA and therefore reduce DNA fragmentation and cell death (Zhang et al., 2016). Moreover, other studies showed that phenolic compounds like flavonoids and phenol acids of fruits have the property of inducing the expression of the P53 protein, a factor that regulates DNA damage repair system preserving the integrity of the DNA (Ahmad et al., 2023). On the other hand, the phenolic compounds of the fruits have the property to modulate at the transcriptional level the expression of the DNA repair protein Poly(ADP-Ribose) Polymerase, a key enzyme involved in DNA repair by base excision (Lagunas-Rangel and Bermudez-Cruz, 2020). 

Despite the high sensitivity in detecting the DNA strand breaks, the comet assay used in this study to evaluate the genoprotective activities of the extracts does not allow the detection of DNA bases mutations which are also a form of genotoxicity. So it does not allow to evaluate the anti-mutagenic properties of the extracts. 

The methanol fraction has exhibited in this study the best DNA protection/repair activities. The methanol fraction would contain antioxidant compounds like flavoinoids and phenol acids which could quench ROS/RNS, thus preserving the integrity of the DNA. The methanol fraction could be used as phytomedicament in the prevention and treatment of the oxidative stress related diseases such as cancer, diabetes, cardiovascular and neurodegenerative disorders. Further investigations should be undertaken to isolate phytomolecules from the methanol fraction and to determine their property of inducing the expression of genes involved in DNA protection/repair.
